# Unveiling Fahr's Syndrome in a Child: A Case Linked to Congenital Hypoparathyroidism

**DOI:** 10.7759/cureus.84001

**Published:** 2025-05-13

**Authors:** Ghizlane Kassal, Rabiy Elqadiri, Soumia Mghar, Houda Nassih, Aicha Abourrahouat, Sara Ouassil, Imane Aitsab

**Affiliations:** 1 Mother and Child Department, General Pediatrics, University Hospital Center, Mohammed VI, Marrakech, MAR; 2 Radiology Department, University Hospital Center, Mohammed VI, Marrakech, MAR

**Keywords:** child, hypocalcemia, hypoparathyroidism, psychomotor delay, secondary fahr´s syndrome

## Abstract

Fahr's syndrome is a rare neurological disorder marked by unusual calcium deposits in specific brain regions. Its occurrence is linked to various underlying causes, including hormonal imbalances, genetic predispositions, infections affecting the central nervous system, and exposure to certain toxic substances.

We describe a pediatric case of Fahr's syndrome linked to hypoparathyroidism, presenting with seizures and developmental delays. Suspicion of this diagnosis was raised due to anamnestic, clinical, and laboratory findings and confirmed after the cerebral computed tomography (CT) scan showed brain calcifications.

Emphasizing the necessity of early identification, this case underscores the value of clinical and biological markers in guiding diagnosis. Additionally, it highlights the essential role of imaging techniques in confirming the presence of brain calcifications and aiding effective management.

## Introduction

Basal ganglia calcifications are linked to a wide range of neurological and metabolic disorders, though they can also frequently appear as incidental findings during neuroimaging of individuals without symptoms [1]. These calcifications have traditionally been classified under the terms Fahr's disease or Fahr's syndrome.

Fahr disease, also referred to as idiopathic basal ganglia calcification (IBGC) or primary familial brain calcification (PFBC), is distinct from Fahr syndrome due to differences in etiology and underlying mechanisms [2-4]. The term "Fahr syndrome" is specifically used when the calcifications in the brain result from a secondary cause that may be reversible or treatable [5]. This syndrome is frequently linked to various medical conditions, with endocrine disorders being particularly prominent. In such cases, metabolic imbalances, especially those affecting calcium and phosphorus homeostasis, can alter the calcium/phosphorus ratio. This disruption leads to the precipitation of colloidal substances in cerebral blood vessels, ultimately resulting in the formation of calcified deposits within the brain [6]. Recent research suggests that hyperphosphatemia promotes osteogenic differentiation, while subnormal parathyroid hormone (PTH) levels suppress anti-osteogenic molecules. Together, these factors create a synergistic effect that accelerates calcification [7].

Fahr's syndrome is a rare neurological disorder with limited cases reported, especially among children. Its pathogenesis, etiology, and treatment remain poorly understood. This report highlights the case of an 11-year-old child with Fahr's syndrome secondary to hypoparathyroidism, presenting with refractory seizures. By sharing this case, the aim is to enhance global understanding of the condition's causes, mechanisms, clinical features, and diagnostic approaches.

## Case presentation

The patient is an 11-year-old boy born to parents in a first-degree consanguineous marriage. The pregnancy was unmonitored, and the delivery occurred at home through a normal vaginal delivery without complications. His medical history reveals psychomotor developmental delay, characterized by intellectual disability, difficulties with walking, speech disorders, and delayed dental eruption. Since the age of six months, he has been under treatment with sodium valproate for generalized tonic-clonic seizures. Additionally, two of his cousins, one maternal and one paternal, are known to be under follow-up for congenital hypoparathyroidism.

The patient presented to the pediatric emergency department with a history of worsening seizures accompanied by recurrent tetany episodes, persisting for approximately three months.

**Figure 1:**
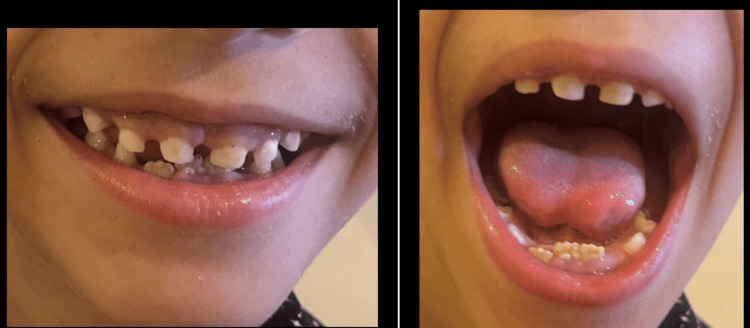
Diastema of both upper and lower arches

Following the stabilization of the patient and the cessation of convulsive seizures, the clinical examination showed normal weight and height but identified key oral manifestations, including delayed dental eruption and poor oral hygiene, as depicted in Figure 1. These images highlight diastema in both the upper and lower arches, further demonstrating the dental abnormalities associated with the condition.

Neurological examination showed a cerebellar syndrome, with an ataxic gait, dysmetria, and dysdiadochokinesia. Psychomotor development was globally delayed, with persistent language impairment and mild cognitive deficits. Muscle tone was normal, and no signs of pyramidal or extrapyramidal involvement were noted. Deep tendon reflexes were preserved and symmetrical. There were no sensory or cranial nerve abnormalities. The patient showed no signs of dysmorphic syndrome or respiratory distress, and the osteoarticular examination revealed no abnormalities.

The biological assessment revealed hypocalcemia, with a total serum calcium concentration of 5.40 mg/dL (normal range: 9.20-10.5 mg/dL), hyperphosphatemia, with serum phosphorus levels at 10.22 mg/dL (normal range: 4.1-5.9 mg/dL), and a decreased intact parathormone (iPTH) level of 4 pg/mL (normal range: 16.2-63 pg/mL). Serum magnesium levels were nearly normal at 1.6 mg/dL (normal range: 1.6-2.5 mg/dL). Additionally, total vitamin D (D2 + D3) levels were slightly reduced at 18.91 ng/mL (normal range: ≥20 ng/mL). A summary of his initial and follow-up laboratory investigations is presented in Table 1.

**Table 1:** Summary of the results of laboratory investigations at admission and follow up ND: Not done, iPTH: intact parathormone

	Admission	1 month after admission	3 months after admission	Reference range
Calcium (mg/dl)	5.4	9.4	9.7	9.2-10.5
Phosphore (mg/dl)	10.6	7.01	6.1	4.1-5.9
iPTH (pg/l)	<4	ND	ND	16.2-63
Calciuria(mg/kg/24h)	3.3	3.6	ND	<4
Vitamin D (nmol/l)	18.5	31.0	45	≥ 20
Magnesium(mg/dl)	1.6	1.9	ND	1.6-2.5

**Figure 2:**
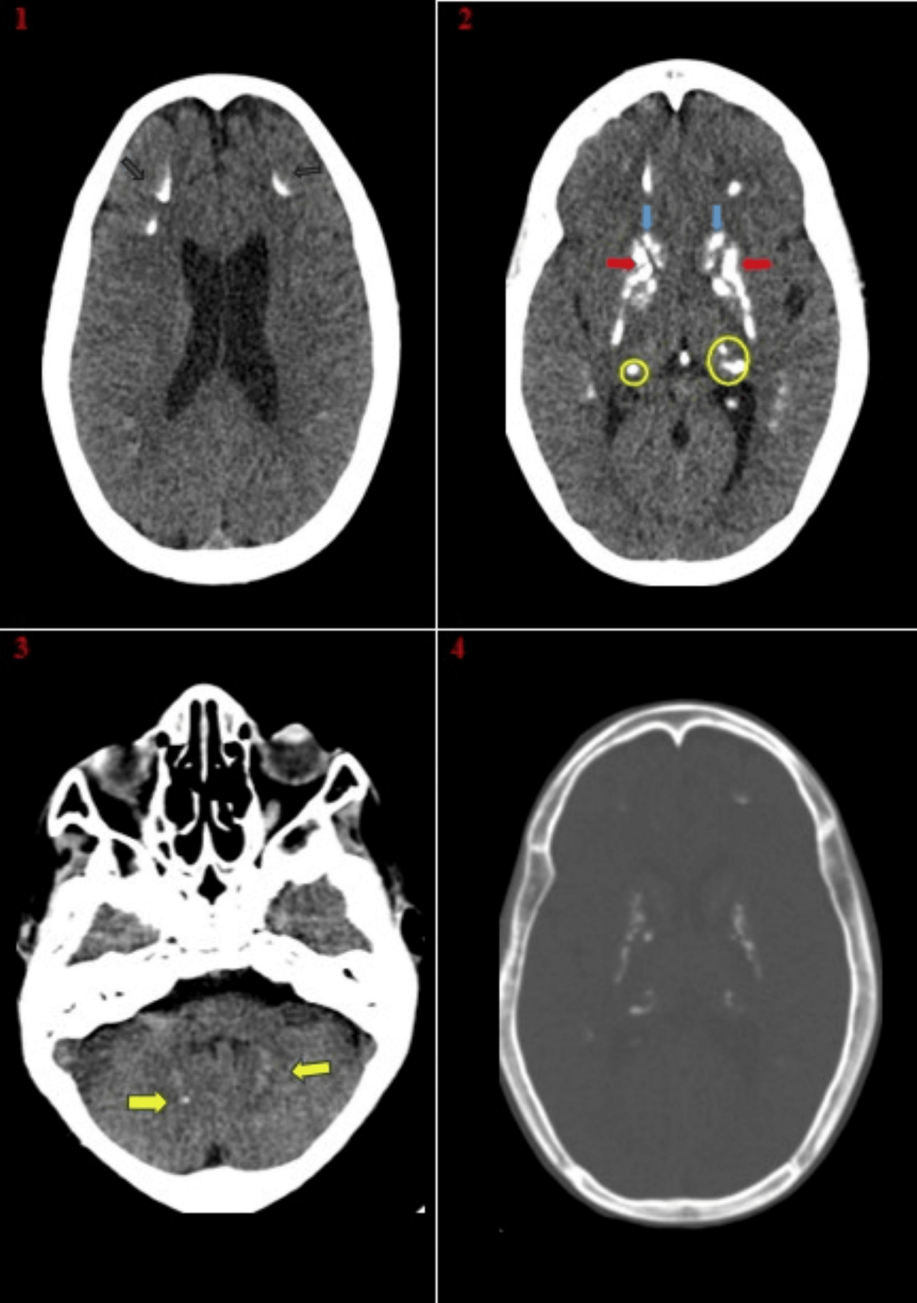
Non-contrast CT of the brain (axial CT images) (1): Arc-shaped calcifications at the junction of gray matter and white matter (arrow). (2): Coarse calcifications localized within the caudate nuclei (indicated by the blue arrow), lentiform nuclei (marked by the red arrow), and pulvinar regions (circled). (3): Fine, low-density calcifications localized within the dentate nuclei of the cerebellum (indicated by the arrow). (4): Non-contrast CT scan using a bone window reveals densities comparable to those of the previously mentioned calcifications.

Brain CT imaging performed at admission revealed symmetrical bilateral calcifications affecting the basal ganglia, deep cerebellar nuclei, and the gray matter-white junction, as shown in Figure 2.

Electroencephalographic monitoring showed no definite interictal epileptiform discharges. Ophthalmological assessment identified bilateral cerulean cataracts, for which surgical intervention was recommended. Meanwhile, electrocardiography, echocardiography, and chest X-ray findings were all unremarkable.

To further investigate the etiology of Fahr’s syndrome and assess other organ involvement, a series of specific tests were conducted. TORCH serologies, immunological assessments, hormonal profiling, thyroid and renal function tests, and protein level analyses all yielded normal results. Thyroid sonography revealed no structural abnormalities, while abdominal-pelvic ultrasound and hearing screening were also normal.

After ruling out potential secondary causes, including infections, metabolic disorders, autoimmune conditions, and vascular diseases, a diagnosis of secondary Fahr’s syndrome associated with congenital hypoparathyroidism was confirmed. Management included intravenous calcium gluconate for immediate correction of hypocalcemia, followed by oral calcium carbonate, alpha-calcidiol, and vitamin D supplementation. Antiepileptic medication was discontinued.

Short-term follow-up showed a favorable biological response with corrected calcium levels, while clinically, seizure activity had ceased. At the three-month follow-up, the patient demonstrated significant improvements, including the absence of seizures, enhanced gait and coordination, improved language skills, and normalization of dental eruption. He is now attending the third year of primary school (CE3) and leading a well-adjusted life.

## Discussion

The relationship between Fahr disease and Fahr syndrome remains a topic of ongoing discussion within the scientific community. Although the terms are often used interchangeably, they are typically classified into two distinct forms: a primary condition known as familial cerebral ferrocalcinosis (PFBC), which lacks an identifiable cause, and a secondary form resulting from an underlying metabolic, infectious, or other medical condition. The term “Fahr disease” is generally used for primary familial brain calcification, whereas “Fahr syndrome” refers to cases with secondary origins [8,9]. 

Secondary brain calcification can occur due to a variety of medical conditions, with an accurate differential diagnosis relying on clinical history, physical examinations, and laboratory findings. Among the recognizable causes, endocrine disorders-such as hypoparathyroidism-disrupt calcium and phosphorus homeostasis, leading to calcifications. Additionally, secondary factors, including infections and toxic exposures, can be identified through a thorough review of documented medical and environmental histories [5,6]. The condition typically manifests in the third or fourth decade of life and is observed twice as frequently in males as in females [8,10].

A prevalent cause of Fahr syndrome is hypoparathyroidism, a disorder in which the parathyroid glands produce insufficient parathyroid hormone (PTH), a crucial regulator of calcium and phosphorus levels in the body. Reduced PTH secretion results in hypocalcemia and hyperphosphatemia, contributing to the development of brain calcifications [11].

The primary causes of calcium homeostasis disturbances include idiopathic and secondary hypoparathyroidism. Idiopathic hypoparathyroidism is particularly rare, with an estimated incidence of 37 cases per 100,000 individuals [11]. It is characterized by the absence, fatty degeneration, or atrophy of the parathyroid glands, leading to decreased PTH secretion and subsequent calcium dysregulation [5].

Permanent hypoparathyroidism causes chronic hypocalcemia, which manifests as a range of clinical symptoms, including paresthesia (tingling sensations), tetany (muscle spasms), a positive Chvostek sign, parkinsonism, extrapyramidal symptoms, cataracts, and QT interval prolongation on electrocardiogram (EKG) recordings. Severe hypocalcemia (below 5 mg/dL) may result in seizures and cardiac arrhythmias [12,13-14]. The imbalance of calcium and phosphorus, particularly from PTH dysfunction, is one of the primary contributors to calcifications in white matter and bilateral subcortical nuclei [15]. In an Indian cohort study comprising 147 patients diagnosed with idiopathic hypoparathyroidism, 107 individuals (73.8%) demonstrated calcifications within the basal ganglia as detected through CT scans. The manifestation and progression of these calcifications were strongly associated with diminished Ca/P ratios [16].

Literature indicates that CT scans commonly display symmetrical bilateral calcifications in regions such as the basal ganglia, thalamus, subcortical white matter, corona radiata, and cerebellum [5,13,17]. Neurological symptoms span a wide range, encompassing seizures, episodes of unconsciousness, falls, difficulties in walking, postural instability, cognitive impairment, parkinsonian features, and resting tremors [13,14]. These neurological symptoms and radiological features align with the observations described in our case report, reinforcing the connection between clinical presentation and imaging findings. Although basal ganglia calcifications are a universal finding in all cases, the clinical presentations exhibit notable variability. This diversity underscores the lack of a direct correlation between the affected brain regions and the spectrum of observed symptoms. To accurately diagnose Fahr’s syndrome, reference to the revised Fahr classification criteria is essential [5].

**Table 2:** Revised diagnostic criteria for Fahr’s syndrome Criteria adapted from Saleem et al. [5], licensed under CC-BY.

Criterion	Description
Bilateral basal ganglia calcification	Confirmed through neuroimaging, with potential involvement of other brain regions.
Progressive neurological dysfunction	Typically manifests as movement disorders and/or neuropsychiatric symptoms. While onset is most common in the fourth or fifth decade of life, cases can also appear in childhood.
Absence of biochemical abnormalities	No somatic markers indicative of mitochondrial/metabolic disease or other systemic conditions.
Exclusion of other causes	No infectious, toxic, or traumatic factors contributing to the condition.
Family history	Autosomal dominant inheritance supporting the genetic basis of the disorder.

 Various treatments have been implemented for Fahr's disease, aiming to achieve either remission or stabilization of the condition. These approaches are based on differing biological theories and limited clinical experiences, often drawing insights from small-scale studies. Timely diagnosis and appropriate treatment of the underlying condition can potentially slow or halt the calcification process. This is exemplified by a documented case where addressing hypoparathyroidism led to the reversal of intellectual disability in a three-year-old child diagnosed with Fahr's syndrome [18].

Seizures and movement disorders linked to Fahr's syndrome, resulting from parathyroid dysfunction, can be alleviated by correcting phosphate and calcium imbalances. For instance, in some studies it has been described that the administration of alpha-hydroxy vitamin D3 and corticosteroids has been proven effective in reversing neurological deficits [19,20].

Currently, there are no specific guidelines available regarding the efficacy of anti-epileptic treatment in resolving seizures in patients with hypocalcemia and cerebral calcifications [21]. A clinical trial investigating the effectiveness of anti-epileptic therapy, conducted on a limited cohort of patients with hypoparathyroidism and epileptic seizures as the initial symptom, demonstrated no significant difference in seizure-free duration between the treatment and non-treatment groups. This lack of efficacy was also observed in the subset of patients with subcortical calcifications. However, seizure suppression appeared to be significantly correlated with the normalization of calcium levels [21].

An alternative therapeutic strategy involving the administration of calcium and 1-alpha-hydroxy-vitamin D3, in conjunction with antiepileptic treatment, was evaluated in a cohort of patients with seizures associated with idiopathic hypoparathyroidism. The metabolic intervention enabled the discontinuation of antiepileptic therapy in approximately 71% of the patients [22].

## Conclusions

Our understanding of Fahr's syndrome has significantly advanced, particularly regarding its etiology, diverse phenotypic manifestations, and improved diagnostic approaches, especially in younger patients. Nonetheless, the complexities and unresolved challenges of this condition persist. There is a pressing need for more comprehensive qualitative and quantitative research to capture the experiences of affected families. Furthermore, establishing an international consensus on care guidelines, incorporating input from all relevant specialties, is crucial for the effective management of both pediatric and elderly individuals living with Fahr's syndrome.

In resource-constrained areas, it is essential for general practitioners to consider rare causes of seizures to prevent complications. In cases of metabolic imbalances, timely diagnosis is essential for addressing electrolyte disturbances, minimizing the unwarranted use of antiepileptic drugs, and preventing their associated adverse effects.
